# Outpatient total hip or knee arthroplasty in ambulatory surgery center versus arthroplasty ward: a randomized controlled trial

**DOI:** 10.1080/17453674.2019.1686205

**Published:** 2019-11-04

**Authors:** Christian Husted, Kirill Gromov, Helle Krogshøj Hansen, Anders Troelsen, Billy B Kristensen, Henrik Husted

**Affiliations:** a Department of Orthopedic Surgery, Copenhagen University Hospital, Hvidovre;; b Ambulatory Surgery Center, Copenhagen University Hospital, Hvidovre, Denmark

## Abstract

Background and purpose — Discharge on the day of surgery (DOS) in selected patients operated with total hip arthroplasty (THA) or total knee arthroplasty (TKA) has been shown to be feasible, but different factors may determine whether patients are discharged on the DOS or not and setting may be one of them. We investigated the importance of the setting in which the short stay following outpatient THA or TKA takes place: was there a difference between the proportion of patients being discharged on the DOS from an ambulatory surgery center (ASC) compared with patients staying on an arthroplasty ward?

Patients and methods — 50 patients (30 TKA, 20 THA) were included in the study and postoperatively randomized to either staying in the ASC or the arthroplasty ward until discharge. All patients were operated under general anesthesia by the same experienced surgeon (HH) and were discharged upon fulfillment of standardized discharge criteria.

Results — 24/25 of the patients who stayed in the ASC compared with 20/25 of the patients on the arthroplasty ward were discharged on the DOS following fulfillment of discharge criteria (p = 0.08). All THA patients were discharged on the DOS and significantly more TKA patients were discharged from the ASC (15/16) vs. from the ward (9/14) (p = 0.04).

Interpretation — Despite fixed discharge criteria, the logistical setting may play a role for achieving discharge on DOS and the ASC may facilitate achieving discharge criteria earlier especially in TKA.

The successful implementation of fast-track hip and knee arthroplasty (THA and TKA) in many departments has resulted in a reduction in perioperative morbidity and mortality, with a concomitant reduction in length of stay (LOS) and a reduction in cost (Andreasen et al. 2017) as functional discharge criteria were achieved earlier. Therefore, standardized outpatient arthroplasty has gained interest in an increasingly competitive financial environment (Argenson et al. 2016, Vehmeijer et al. 2018). Fast-track is based on clinical and logistical optimization via identification of clinical and logistical barriers to overcome (Husted et al. 2011, Husted 2012). Accordingly, implementation of outpatient arthroplasty requires monitoring of safety, patient satisfaction, and economic impact.

A various number of unselected THA and TKA patients are eligible for outpatient surgery depending on underlying demographics and comorbidities of the specific population. Though many patients undergo surgery in such a setting, the proportion of patients who are discharged on the day of surgery (DOS) as intended varies substantially from around 25% (Gromov et al. 2017) to 75–99% (Berger et al. 2009, Chen and Berger 2013, Hartog et al. 2015, Parcells et al. 2016, Goyal et al. 2017). Since no reports of consistent 100% discharge on DOS have been published, back-up allowing overnight stays for medical/surgical complications is considered mandatory by some (Crawford et al. 2019).

While patient selection clearly plays a role in successful DOS discharge following THA and TKA, the logistical set-up may also influence the number of patients fulfilling the discharge criteria on DOS (DeCook 2019).

This RCT examines whether outpatient surgery and subsequent postoperative stay at an ambulatory surgery center (ASC) improved discharge on DOS compared with a postoperative stay on an arthroplasty ward.

## Patients and methods

**Figure 1.**
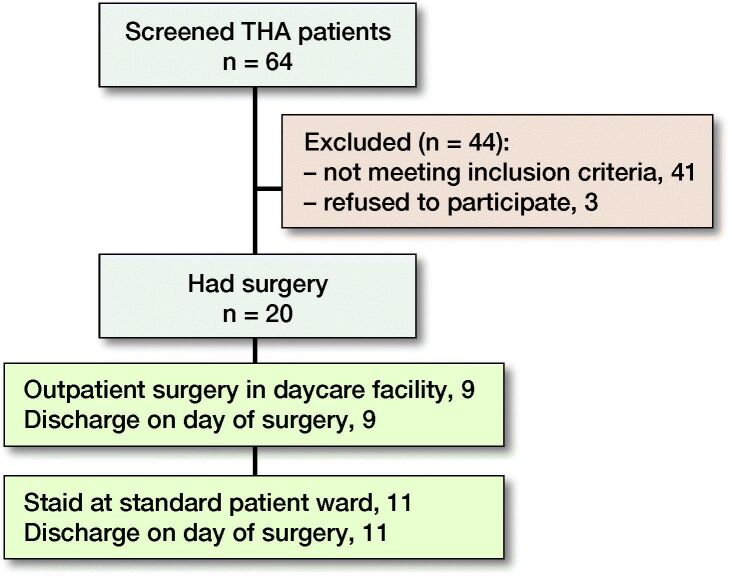
Flow chart of included THA patients.

**Figure 2.**
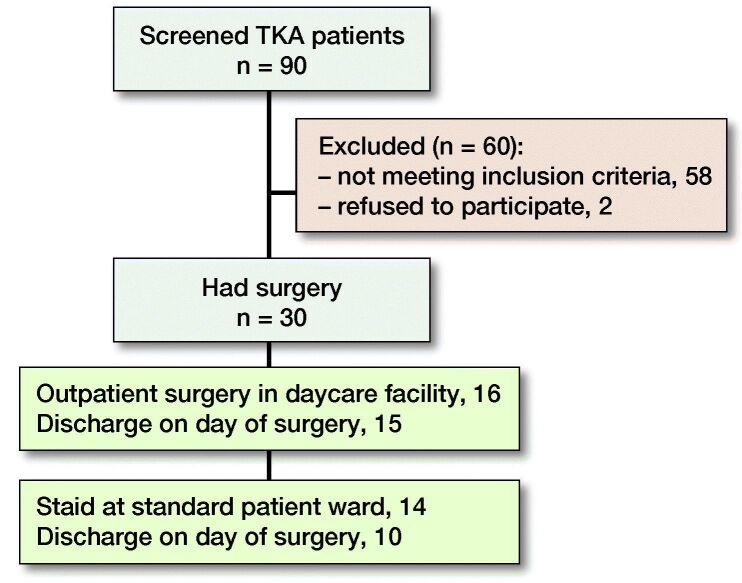
Flow chart of included TKA patients.

The CONSORT guidelines were followed. 154 patients (64 THA, 90 TKA) were screened for eligibility to have fast-track surgery with the intent of discharge on the DOS. Of those, 50 patients (33%) (20 THA, 30 TKA) were included in the study based on the inclusion criteria (Table 1, Figures 1 and 2). All 50 surgeries were done by the same experienced surgeon (HH) in the ASC between August 2016 and November 2018. Patients received information concerning the surgical procedure and intent of discharge on DOS provided there was fulfillment of the discharge criteria (Table 2).

**Table 1.** Inclusion criteria

•Patients with clinical and radiological osteoarthritis of the hip suitable for primary cementless THA and patients with clinical and radiological osteoarthritis of the knee suitable for primary CR TKA
•Age 18–80
•ASA < 3
•Interested in and motivated for discharge on DOS
•Family/relatives to be present for > 24 hours after discharge
•Able to understand and give consent to the study

**Table 2.** Discharge criteria

•Activity level: Steady gait with crutches, no dizziness. Stairs if required
•Nausea and/or vomiting: Minimal and efficiently treated with or without medications
•Vital signs: Must be stable and consistent with age and preoperative baseline. Systolic blood pressure within 20 mmHg of preoperative levels. Saturation > 95%. Pulse < 100 while resting
•Pain: The level of pain that the patient has should be acceptable to the patient. VAS < 3 at rest and VAS < 5 on mobilization
•Surgical bleeding: Postsurgical bleeding should be consistent with expected blood loss for the procedure and not require repeated dressing change. Patients should be hemodynamically stable (no tachycardia (pulse >100 at rest) and hypotension sBP < 100) and show no clinical signs of anemia (paleness, dizziness during mobilization and fatigue).

1 week before surgery patients were informed and instructed on the upcoming procedure by the same experienced anesthesiologist (BBK) and physiotherapist.

Patients received 400 mg of celecoxib and 1 g of paracetamol on the morning of surgery. A single intraoperative dose of 125 mg of methylprednisolone was administered IV. General anesthesia was achieved by 2–3 mg/kg of IV propofol and 0.5 µg/kg/min remifentanil. A laryngeal mask was used for airway management and no oxygen was given during induction. Continuous infusion of propofol 10 mg/mL, 4–6 mg/kg/h and remifentanil 2 mg, 0.25–0.5 µg/kg/min was used to maintain anesthesia. Normothermia was maintained through forced air warming. Fluid loss during surgery was replaced with 0.9% saline, 15 mL/kg/h. All THAs were performed using a standard posterolateral approach with simple posterior soft-tissue repair. Infiltration anesthesia (LIA) was not used in THA’s. All TKAs were performed using a standard medial parapatellar approach without the use of tourniquet. Measured resection technique was used with cutting guides applied externally on the tibia and intramedullary on the femur and application of local infiltration analgesia (LIA, 150 mL Ropivacaine). No drains were used for any surgery. Postoperative radiographs were obtained in the operating room, approved by the surgeon and handed out to the patient.

Until discharge and in case of VAS > 50 mm at rest patients were given rescue analgesics consisting of sufentanil 5–10 µg IV or 10 mg of oral morphine. Postoperatively, 200 mg/12 h of celecoxib and 1 g of paracetamol/6 h were administered up to and including postoperative day 6. Furthermore, 10 tablets of 10 mg opioid (morphine) were given to the patient to use at home if needed. Further pain management was handled by the patients’ general practitioners. Postoperative nausea and vomiting were treated with 4 mg of ondansetron. Oral thromboprophylaxis consisting of rivaroxaban was started 6–8 hours after surgery and continued for 2 days. No mechanical thromboprophylaxis or extended oral thromboprophylaxis was used. Physiotherapy was started as soon as possible after surgery and focused on achieving unassisted gait with crutches.

Immediately after surgery in the ASC the patients were randomly selected (opaque numbered envelopes not discriminating between THA or TKA) either to be taken to the arthroplasty ward or to stay in the ASC.

Patients meeting discharge criteria before 8 pm were discharged to their own homes (Table 2). Patients staying in the ASC not meeting the discharge criteria prior to 8 pm were transferred to the arthroplasty ward for overnight stay. All patients were discharged to their own homes.

### Statistics

Prior to the study, a pilot series of 20 patients were operated at the ASC with 95% discharged on the DOS. As 24–28% were discharged from the arthroplasty ward in a previous study (Gromov et al. 2017) and we estimated that number could double (60%) with the focus of the study, a power calculation using power 0.8, alpha 0.05, beta 0.2 found 2 x 21 patients to be needed to show a statistical difference. Thus, we included 25 patients in each arm to account for potential dropout.

The Pearson chi-Square test and the independent samples t-test were used to compare data. Data was tested for normality using the Shapiro–Wilk test. A statistically significant difference between two sets of comparable data was defined as p < 0.05. All statistical analyses were performed in IBM SPSS Statistics 25 (IBM Corp, Armonk, NY, USA).

### Ethics, registration, funding, and potential conflicts of interest

No approval from the National Ethics Committee was necessary (study protocol presented and waived), as this was a non-interventional observational study. The study was approved by the Danish Data Protection Agency (registration no. 2007-58-0015) and registered with ClinicalTrials.gov (Identifier: NCT03896282). There was no funding and no conflict of interest.

## Results

On the DOS 44 of the 50 patients were discharged, then 5 patients 1 day after surgery and 1 patient 2 days after surgery.

According to the randomization, 11/20 THA and 14/30 TKA patients were transferred to the arthroplasty ward after surgery and 9/20 THA and 16/30 TKA patients stayed at the ASC after surgery. All 20 THA patients were discharged on DOS and 24/30 of the TKA patients were discharged on the DOS. 9/14 of the TKA patients who were transferred to the arthroplasty ward after surgery were discharged on DOS compared with 15/16 of the TKA patients staying at the ASC. All patients who were not discharged directly from the ASC were transferred to the arthroplasty ward and discharged home on the next day.

Between patients staying at the ASC and the patients at the arthroplasty ward, sex, age, ASA score, BMI, surgery time, blood loss, and Oxford Hip Score prior to surgery was similar (Table 3). A statistically significant difference between the 2 groups of patients was found regarding Oxford Knee Score pre-surgery indicating that patients at the ASC had less pain and better function prior to surgery.

**Table 3.** Demographics, numbers, and statistical significance

	Ambulatory	Arthroplasty	
TKA patients	surgery center	ward	p-value
Male, n (%)	8	5	
Female, n (%)	8	9	
BMI, mean (SD)	28 (4.2)	29 (4.5)	
Age (years), mean (SD)	58 (7.7)	63 (10.1)	
ASA score 1, n (%)	8	4	
ASA score 2, n (%)	8	10	
Surgery time (min), mean (SD)	56 (11)	53 (9)	0.5
Blood loss (mL), mean (SD)	209 (78)	179 (77)	0.3
OKS, mean (SD)			
pre-surgery)	26 (6)	22 (7)	0.05
3 months post-surgery	35 (5)	29 (9)	0.03
ΔOKS	9 (9)	7 (9)	0.7

	Ambulatory	Arthroplasty	
THA patients	surgery center	ward	p-value
Male, n (%)	4	5	
Female, n (%)	5	6	
BMI, mean (SD)	25 (5)	26 (5)	
Age (years), mean SD	58 (8.8)	60 (7.2)	
ASA score 1, n (%)	6	9	
ASA score 2, n (%)	3	2	
Surgery time (min), mean (SD)	61 (12)	54 (8)	0.1
Blood loss (mL), mean (SD)	272 (218)	272 (154)	1.0
OHS, mean (SD)			
pre-surgery	24 (4)	21 (3)	0.1
3 months post-surgery	41 (3)	38 (7)	0.3
ΔOHS	17 (5)	17 (6)	0.9

Oxford Knee and Hip Scores were also recorded 3 months after surgery and compared with the preoperative scores. No statistically significant difference was found in the progression of these scores between the two groups (Table 3).

24/25 of all the patients who stayed at the ASC were discharged on the DOS compared with 20/25 of the patients on the arthroplasty ward (p = 0.08). All THA patients were discharged, but significantly more TKA patients were discharged from the ASC (15/16) vs. from the ward (9/14) (p = 0.04).

3 weeks after surgery, TKA patients had their staples removed and pain VAS scores were assessed at rest and during activity with weight-bearing on the knee. Among TKA patients who stayed on the arthroplasty ward following surgery, a mean VAS score of 2.6 (SD 1.6) was recorded at rest whereas the average VAS score among TKA patients staying at the ASC was 1.4 (1.2) at rest (p = 0.06). During weight-bearing activity TKA patients from the arthroplasty ward had a mean VAS score of 3.4 (1.5) compared with a mean VAS score of 2.3 (1.6) among TKA patients from the ASC (p = 0.09).

The THA patients had their staples removed 2 weeks after surgery and pain VAS scores at rest and activity were also recorded here. At rest, THA patients from the arthroplasty ward had a mean VAS score of 2.3 (1.8) whereas THA patients from the ASC had a mean VAS score of 1.4 (1.4) (p = 0.3). During activity, a mean VAS score of 3.3 (1.6) was recorded among THA patients from the arthroplasty ward compared with 2.6 (1.5) within the group of THA patients from the ASC (p = 0.4).

## Discussion

In this single-center randomized controlled trial, we found more patients being discharged on the DOS when staying in the ASC compared with patients staying on the arthroplasty ward following identical discharge criteria. Since all THA patients were discharged on the DOS, the difference in proportion of same-day discharge between the ASC and the ward is exclusively due to TKA patients. Logistical factors that could influence LOS—and hence specifically ability to discharge on the DOS—have been studied and include weekday of surgery and surgical start time on the DOS (Husted and Holm 2006, Keswani et al. 2016, Boylan et al. 2017) as well as bypassing the post-anesthesia care unit facilitating earlier functional rehabilitation (Lunn et al. 2012). Also, the specific setting, including the location immediately after surgery (single bed, multiple beds in same room, open area like ASC), type of bed (regular hospital bed or recovery bed) and the staffing, including immediate availability of anesthesiologic assistance, may be of importance. Hence, we studied the importance of a set-up in the ASC where patients are lying in recovery beds in an open space with full visibility, nurses around them and an anesthetist present. This was compared with a traditional arthroplasty ward set up for fast track during more than 15 years and familiar with outpatient arthroplasty (Gromov et al. 2017) where the patients are lying in beds confined to their room with other inpatients around them, nurses not within sight in the room (but on call immediately outside) and no anesthesiologist present.

Several factors may explain the difference in same-day discharge ratio. A dissimilarity in motivation for discharge on the DOS between the 2 patient groups seems plausible. Patients staying on the arthroplasty ward following surgery shared the ward with inpatients who were not planning on same-day discharge as opposed to patients in the ASC who were surrounded by fellow outpatients from different specialties all intended to be discharged within a few hours. This difference may induce psychological priming to same-day discharge.

On the arthroplasty ward, patients were staying in regular beds with pillows and duvets whereas recovery beds were used at the ASC. While the former may not encourage patients to get out of bed, the recovery beds, being less comfortable, were also tipped in anti-Trendelenburg very early, mimicking sitting and standing positions and thereby improving hemodynamics and trying to overcome orthostatic intolerance.

Differences between staff/patient ratio on the arthroplasty ward and the staff at the ASC could also be part of the explanation as the latter is more staff-intensive allowing more focus on each patient but at a higher cost (Husted et al. 2018). This could affect the efficiency of management of pain, nausea, and dizziness as the staff on the arthroplasty ward have more patients to look after and therefore cannot administer medicine as quickly to patients in need of it. Also, in the ASC, a dedicated anesthesiologist is monitoring the patients closely postoperatively resulting in faster and more efficient management of pain, nausea, and dizziness, which ultimately ensures earlier fulfilment of the fixed functional discharge criteria.

The presence of the anesthetist was indeed a major difference, which reflects the nature of the set-up in the ASC. Where both groups were treated similarly re pain medication (paracetamol, celecoxib, methylprednisolone, LIA in TKA, general anesthesia) and had access to the same standard medication re pain reduction when needed (given by the nurses in both locations), the anesthetist could be consulted if needed in the ASC. He could intervene earlier regarding gaining sufficient pain control or treat dizziness believed to be due to orthostatic intolerance (ephedrine). However, no recording of the amount of consulting or the need for intervention by the anesthetist was performed as the study aim was simply to illuminate whether the different set-ups including the presence of a dedicated anesthetist in the ASC influenced the number of patients who could be discharged on the day of surgery.

All the differences above in the setting probably contribute to the increased number of TKA patients able to be discharged on the DOS. As THA is an operation with less surgical stress response and less postoperative pain compared with TKA (Andersen et al. 2009), it seems that the specific setting has less importance for this group of patients, which may also at least partly explain the higher incidence of same-day discharge in THA patients (Hartog et al. 2015).

As no other study has focused on the potential importance of different set-ups, future studies are necessary in order to define which factors are of specific importance, including an economic evaluation, as same-day surgery is associated with very low cost provided there is no increase in readmissions (Husted et al. 2018).

There were no mortalities, no strokes, no myocardial infarctions, no pulmonary embolisms, and no deep venous thrombosis within 90 days after surgery among all patients. These findings are in line with the previous fast-track findings of very low mortality and morbidity, especially regarding thromboembolic complications (Husted et al. 2010, Jørgensen and Kehlet 2016, 2017) which contributed to the early mobilization perfected in outpatient arthroplasty (Gromov et al. 2019).

It has previously been established that fast-track surgery leads to reduced pain compared with regular joint care protocols (Fransen et al. 2018) but very limited literature is available on the differences in pain between inpatients and outpatients subsequent to total joint arthroplasty (Goyal et al. 2017). In this study patients had VAS scores recorded post-surgery. For the TKA patients, nearly statistically significantly less pain at rest and upon activity was found after 3 weeks in the ASC group. These differences in pain could be attributed to the fact that the TKA patients from the ASC also had better OKS prior to surgery although it could also be a result of better pain management in the early hours after surgery. Since pain scores were lower for THA patients from the ASC (not significantly though), further studies are needed to determine this and early recovery trajectories including pain scores are important measurements (Klapwijk et al. 2017, Porsius et al. 2018).

A strength of this study arises from the standardized surgical, anesthetic, and analgesic regime for all patients. The study might be under-powered to demonstrate potential differences between the groups for secondary outcomes, and it was not powered regarding evaluation of safety aspects.

This study has several limitations. The number of patients was sufficient according to the power calculation and the conservative estimation of twice as many patients being discharged from the arthroplasty ward, but it turned out that 3 times as many patients were discharged on the DOS. Hence, nearly statistically significant differences may have become significant if more patients had been included. Although operation, anesthesia, pain protocol, and physiotherapy were identical, the different settings include a multitude of differences and hence it is impossible to differentiate which specific parameter is paramount. The staff on the arthroplasty ward may have focused more on discharge on the DOS compared with earlier studies as a consequence of the competitive setting.

Staff experience/expectation may indeed influence the likeliness of discharge, even though the universal use of strictly functional discharge criteria may overcome this. We tried to take that into account by ensuring experience with the same-day procedure in both locations by running a pilot series, also in the ASC, before starting and by doubling the expected number of patients able to be discharged from the ward on the day of surgery. Further studies should focus on evaluating all the potential differences between the set-ups in order to find the best cost–benefit set-up for both patients and hospital, which should also include measurement of patient satisfaction with the stays in the 2 different set-ups.

Another limitation could be the pooling of THA and TKA patients as it seems that THA patients are less sensitive to the setting. However, we did not find such a difference in earlier studies (Gromov et al. 2017). Finally, the external validity may be questioned as the findings in this study may be unique for these specific settings.

In summary, more TKA patients were discharged on the DOS when staying at the ASC compared to patients staying on the arthroplasty ward. Therefore, setting appears to play a role when it comes to successful discharges on the DOS among outpatients undergoing THA and TKA. The reasons for this may be manifold and include use of recovery beds, nurse/patient ratio, visibility of staff, and other patients being discharged, as well as the presence of an anesthetist to reduce pain, nausea, and dizziness immediately. Pain trajectories may be improved by this for the first few weeks. These findings require further investigation in order to establish reasons of clinical relevance. The multidisciplinary approach has always been emphasized in fast-track arthroplasty with overnight stay (Husted 2012). but may be even more pronounced in outpatient surgery where the role of the anesthetist becomes especially evident (Oosterholt et al. 2017).
